# P-1994. Rapid COVID-19 Transmission and Multi-layered Interventions in a High-rise Detention Center from January to March 2022 in South Korea

**DOI:** 10.1093/ofid/ofae631.2152

**Published:** 2025-01-29

**Authors:** Jin Su Song, Pyoeng Gyun Choe, Wan Beom Park, Shin Young Lee, Seonju Yi

**Affiliations:** Seoul National University College of Medicine, Seoul, Seoul-t'ukpyolsi, Republic of Korea; Seoul National University Hospital, Seoul, Seoul-t'ukpyolsi, Republic of Korea; Seoul National University College of Medicine, Seoul, Seoul-t'ukpyolsi, Republic of Korea; Korea Diseases Control and Prevention Agency, Sejong-si, Kyongsang-namdo, Republic of Korea; Korea Disease Control and Prevention Agency, Seoul, Seoul-t'ukpyolsi, Republic of Korea

## Abstract

**Background:**

Correctional and detention facilities face significant challenges in controlling the spread of highly infectious pathogens such as SARS-CoV-2 due to overcrowded conditions, inadequate ventilation, and shared facilities. Despite reports on COVID-19 outbreaks in various correctional settings worldwide, investigations into outbreaks in metropolitan high-rise prisons remain scarce, warranting detailed examination.

Diagnosed cases of coronavirus diseases 2019 (COVID-19) in detention center (blue) and South Korea (yellow), January through March 2022

**Figure:**
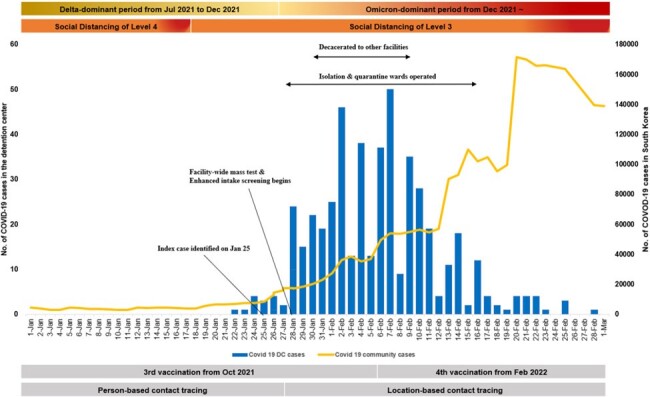

**Methods:**

The detention center, which covers an area of 41,658 mm^2^, can accommodate 2,070 individuals and housed 1,977 inmates and 674 correctional officers at the time of outbreak detection. The facility comprises five buildings, including one 10-story and four 12-story buildings, with 47 wards. Each building is connected with the following building through a common hallway located at the distal end of each building. Descriptive analyses were conducted on demographic characteristics, symptom onset dates, comorbidities, vaccination status, and exposure history of confirmed COVID-19 cases. Vaccine effectiveness was estimated using logistic regression models.

Epidemic curve of SARS-CoV-2 case-patients by building and floor(vertical axis), South Korea, 2022

**Figure:**
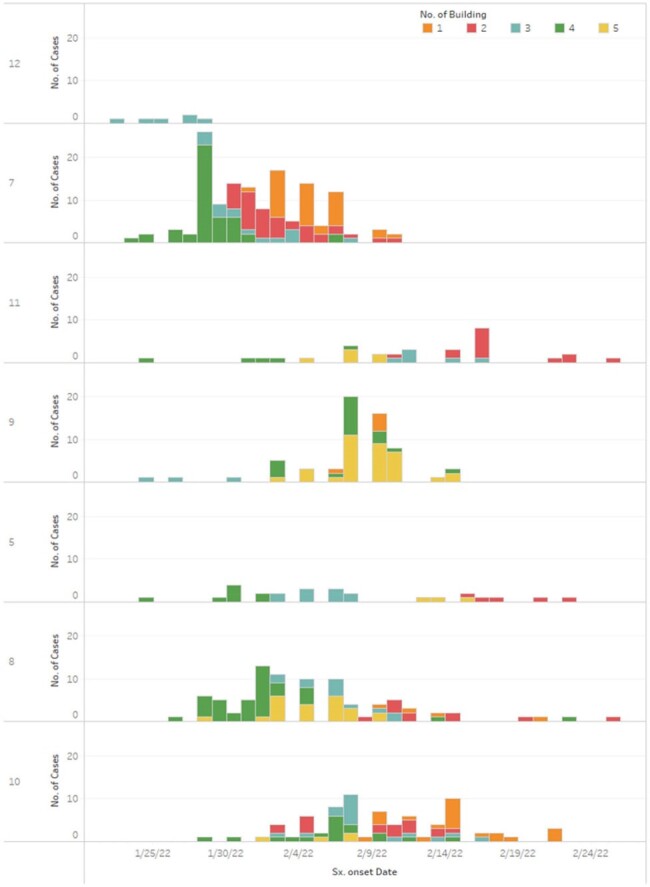

**Results:**

From January 25 to March 2, 2022, 492 people, including 474 (30.0%) of 1,977 incarcerated persons (cumulative attack rate of 24%) and 18 (2.7%) of 674 staff members at the detention center, were confirmed with SARS-CoV-2 infection. High-rise facility characteristics contributed to rapid transmission, with the index case identified on January 25, 2022. However, the implementation of physical distancing, decarceration, limiting movement, and conducting serial and mass testing, despite being complex and requiring substantial resources, effectively slowed the spread of the Omicron variant amidst persistent community transmission. Vaccine effectiveness against infection was estimated at 52.7% (95% confidence interval, 37.2% to 64.4%) for three doses.

Characteristics of coronavirus disease 2019 case-patients in a detention center, South Korea, 2022

**Figure:**
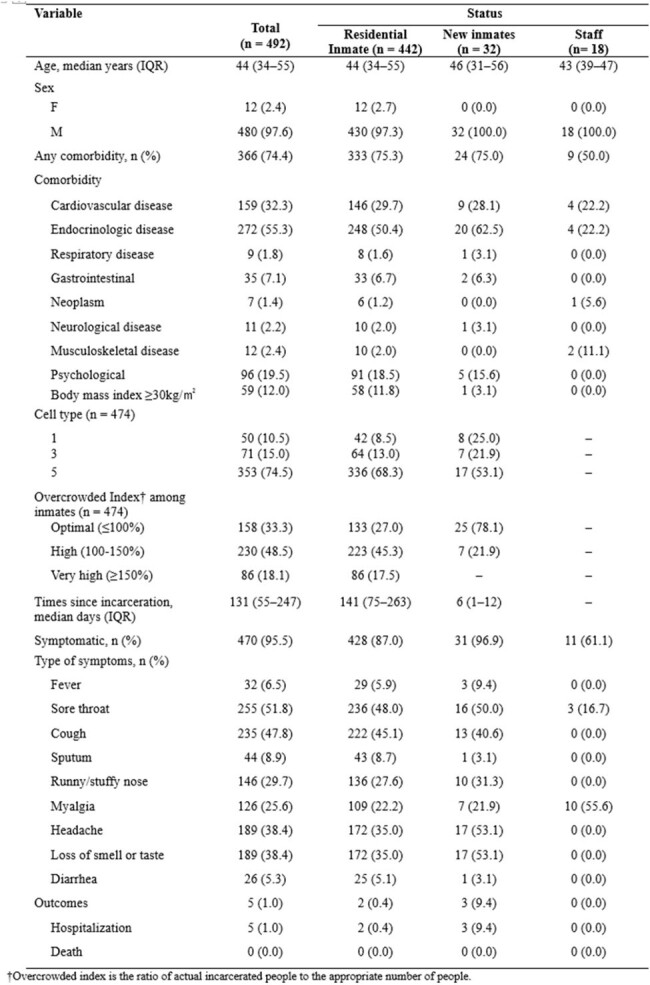

**Conclusion:**

The findings underscore the vulnerability of high-rise detention centers to COVID-19 outbreaks and emphasize the effectiveness of multi-layered interventions in curbing transmission. Swift and comprehensive measures are crucial for controlling outbreaks in high-density, enclosed settings, informing future pandemic response efforts and policy development.

**Disclosures:**

All Authors: No reported disclosures

